# Systematic Evaluation of “Compliance” to Prescribed Treatment Medications and “Abstinence” from Psychoactive Drug Abuse in Chemical Dependence Programs: Data from the Comprehensive Analysis of Reported Drugs

**DOI:** 10.1371/journal.pone.0104275

**Published:** 2014-09-23

**Authors:** Kenneth Blum, David Han, John Femino, David E. Smith, Scott Saunders, Thomas Simpatico, Stephen J. Schoenthaler, Marlene Oscar-Berman, Mark S. Gold

**Affiliations:** 1 Department of Psychiatry and McKnight Brain Institute, University of Florida, College of Medicine, Gainesville, Florida, United States of America; 2 Department of Clinical Neurology, PATH Foundation NY, New York, New York, United States of America; 3 Department of Management Science and Statistics, University of Texas at San Antonio, San Antonio, Texas, United States of America; 4 Meadows Edge Recovery Center, North Kingstown, Rhode Island, United States of America; 5 David Smith and Associates and Institute of Health and Aging, University of California San Francisco School of Medicine, San Francisco, California, United States of America; 6 Dominion Diagnostics, LLC, North Kingstown, Rhode Island, United States of America; 7 Human Integrated Services Unit University of Vermont Center for Clinical and Translational Science, and Department of Psychiatry, UVM College of Medicine, Burlington, Vermont, United States of America; 8 Department of Sociology, California State University, Turlock, California, United States of America; 9 Departments of Psychiatry, Neurology, and Anatomy and Neurobiology, Boston University School of Medicine, and Department of Veterans Affairs Healthcare System, Boston, Massachusetts, United States of America; Penn State College of Medicine, United States of America

## Abstract

This is the first quantitative analysis of data from urine drug tests for compliance to treatment medications and abstinence from drug abuse across “levels of care” in six eastern states of America. Comprehensive Analysis of Reported Drugs (CARD) data was used in this *post-hoc* retrospective observational study from 10,570 patients, filtered to include a total of 2,919 patients prescribed at least one treatment medication during 2010 and 2011. The first and last urine samples (5,838 specimens) were analyzed; compliance to treatment medications and abstinence from drugs of abuse supported treatment effectiveness for many. Compared to non-compliant patients, compliant patients were marginally less likely to abuse opioids, cannabinoids, and ethanol during treatment although more likely to abuse benzodiazepines. Almost 17% of the non-abstinent patients used benzodiazepines, 15% used opiates, and 10% used cocaine during treatment. Compliance was significantly higher in residential than in the non-residential treatment facilities. Independent of level of care, 67.2% of the patients (n = 1963; *P*<.001) had every treatment medication found in both first and last urine specimens (compliance). In addition, 39.2% of the patients (n = 1143; *P*<.001) had no substance of abuse detected in either the first or last urine samples (abstinence). Moreover, in 2010, 16.9% of the patients (*n* = 57) were abstinent at first but not at last urine (deteriorating abstinence), the percentage dropped to 13.3% (*n* = 174) in 2011; this improvement over years was statistically significant. A longitudinal analysis for abstinence and compliance was studied in a randomized subset from 2011, (n = 511) representing 17.5% of the total cohort. A statistically significant upward trend (*p* = 2.353×10^−8^) of abstinence rates as well as a similar but stronger trend for compliance ((*p* = 2.200×10^−16^) was found. Being cognizant of the trend toward drug urine testing being linked to medical necessity eliminating abusive screening, the interpretation of these valuable results require further intensive investigation.

## Introduction

### The issue

Substance use disorders (SUDs) present a formidable challenge to treatment providers. Multi-faceted factors influence the course of the disorder, treatment outcomes and relapse. Evaluation and quantification of these factors is essential to reduce morbidity and maximize positive outcomes. Most clinicians would agree that compliance to prescribed treatment medications, as well as, patients being abstinent from drugs of abuse during treatment, are important outcome challenges in chemical dependence programs. Surprisingly, a 1-17-14 PUBMED search resulted in only one article that matched the following terminology: “urine analysis and compliance to prescribed treatment medications during *in-patient* and or *out- patient* treatment.” A shorter word search did not find any additional articles. One article was found about non-cancer pain patients and the authors concluded: “Regular urine drug testing should be a part of acute and chronic pain management whether or not the patient has any signs or symptoms of drug misuse” (abuse) [1]. Likewise, a 1-17-14 PUBMED search found only four articles that match the following terminology: “urine analysis and abstinence to drugs of abuse during *in-patient* or *out-patient* treatment.” While it is believed by some that compliance of treatment medications in patients undergoing treatment has been studied for over 20 years the present article is the only one to evaluate this topic systematically for both compliance to treatment medications and abstinence of licit and illicit drugs during treatment in one sample analysis.

These articles [2]–[18] were specific to type of intervention and drug of choice but not generalized to the level of care per se or to an entire American state [1], [19]–[21]. While the Institute of Medicine have reviewed information linked to both compliance and abstinence related to drug abuse there is no definitive reported outcome published in the literature to date [6-17-13]. There is one report by Starrels *et al.* [22] that reviewed the literature relating opioid abuse and urine testing and concluded: “Relatively weak evidence supports the effectiveness of opioid treatment agreements and urine drug testing in reducing opioid misuse (abuse) by patients with chronic pain.”

Moreover, a review of the overall literature on “addiction” per se revealed that there are 42,162 PUBMED listed articles [7-23-14]. An exhaustive word search and direct analysis of all the papers including an arduous review of a subset of 50 free articles resulted in only one paper concerning Long-acting Methadone (LAAM) utilizing urine analysis that evaluated both compliance and abstinence simultaneously [23]. Additionally, careful review of the largest cohort of approximately 10,000 patients derived from the national Drug Abuse Treatment Outcome Studies (DATOS), found no evidence of coupling both abstinence and compliance during treatment dependent upon level of care. One study by Simpson *et al.* [24] evaluating cocaine abusing patients based on urine analysis found higher severity of patient problems at program intake and shorter stays in treatment (<90 days) were related to higher cocaine relapse rates.

None of the above studies, however, including Simpson *et al.* [24] evaluated the following important question. When each urine sample measures both prescription medication and illicit drug use of patients being treated for drug addiction, are those who comply with prescribed treatment (as measured in baseline urine) more likely to be in remission at the end of treatment (as measured in urine) than patients who did not comply with prescription treatment (as measured in baseline urine)? The treating physician wants to know for each client as treatment commences if ordering urine tests on both prescription medication and illicit drugs is important, for diagnosis and management and are these tests based upon medical necessity criteria.

Based on the paucity of real quantitative data revealed by our systematic review of the literature, we embarked on this comprehensive study. In terms of clinical importance we are cognizant that clinicians want to see the main findings broken down for, each type of drug use, and each type of prescription treatment, each type of setting (residential, outpatient, etc) and by age and gender. Our data herein provides some of these answers although more analysis and research is required.

### Importance of Urine Drug Testing

Our clinical experience suggests that randomized urine testing is a crucial part of any successful treatment program and as such deserves consideration. According to DuPont and Gold [25] the dominant characteristic of SUDs, is relapse. One of the lessons learned by DuPont and Skipper [26] was that long-term frequent random urine drug and alcohol testing dramatically reduced relapse in physicians mandated to participate in the physician health program (PHPs). Although partly a reflection of the cohesive nature of the program, only 22% of physicians tested positive at any time during the program and at the five-year point 71% were still licensed and employed [25], [27]. The Federal Center for Substance Abuse Treatment also strongly advocates drug urine testing [28]. In fact, Tenore [29] and others [26] emphasized urine toxicology screening as an important standard of care in addiction and pain-treatment settings.

We are cognizant of the trend toward drug urine testing being linked to medical necessity eliminating abusive screening and do not endorse this seemingly *post hoc* abusive screening.

Comprehensive drug monitoring tools have recently been developed to assist clinicians demonstrate precision, at intake, during treatment and for individual and program outcomes [30]. One such clinical tool is the “Comprehensive Analysis of Reported Drugs” (CARD™) a reporting system that uses laboratory results from validated urine drug testing profiles of commonly abused illicit and prescription drugs, their metabolites, biological markers and commercial adulterant screening (that includes: creatinine, specific gravity, pH, and oxidants) and compares them to prescribed and self -reported drug use.

The two key clinical issues during in-patient or out-patient recovery programs are patients' compliance to prescribed treatment medications and patients' abstinence from all non-prescribed licit or illicit psychoactive drugs.

### Monitoring compliance

When combined with counseling and other behavioral therapies, medications are an essential treatment element for many patients with SUD.

Adherence has been defined as, the extent to which a person's behavior conforms to medical or health advice [31]. While there is no data available on compliance to typical Food and Drug Administration (FDA) approved drugs for SUD, Chong *et al.* [32] following an extensive review of the literature, suggested that non-adherence or non-compliance with anti-depressant treatment is very common. Four meta-analyses [33]–[36] have demonstrated that for tricyclic antidepressants and selective serotonin reuptake inhibitors, drop-out rates are in the range of 21–33%. Many psychiatrists and addiction treatment professionals agree that patients attending programs for SUD may likewise not comply with prescribed treatment medications. A literature search revealed a paucity of information regarding this pivotal clinical issue. None of the papers we identified on the subject, used data from biological markers to determine compliance to treatment medications.

### Monitoring abstinence

A further review (1-17-14) of the literature [MEDLINE, PsycINFO, EMBASE, Cochrane Central Register of Controlled Clinical Trials] utilizing the term “monitoring drug abuse abstinence by urine analysis” resulted in fifteen citations regarding abstinence. While most of the studies evaluated drugs like alcohol, opioids and psychostimulants individually, none systematically analyzed abstinence data according to state by state outcomes, by treatment modality, as a function of level of care, or trends over time.

It is noteworthy that without data to measure and control for patient characteristics, including severity of drug use and medical and social problems, such comparisons while important must be interpreted with caution and require more intense investigation.

**Hypotheses:** Two null hypotheses form the basis of this retrospective *post hoc* study. Patients in chemical dependency programs in America: 1) adhere to prescribed treatment medications and 2) abstain from illicit drug use during treatment.

## Methods

### Comprehensive Analysis of Reported Drugs (CARD)

This is a statistical analysis of unidentifiable data from CARD, privately held at Dominion Diagnostics LLC North Kingston RI, used to evaluate treatment adherence in a large clinical cohort from across a number of eastern states in America. Prior to being accessed for this statistical analysis the data was de-identified by Scott Saunders, MS. The ethics committee from Path Foundation NY on November 29th 2012 waived the approval of an IRB for this post hoc retrospective study of unidentifiable data, as well as the need for individual consent by patients to use the database for research. The non-public anonymized data can be provided to researchers, with prior written approval, by Dominion Diagnostics LLC. A detailed explanation of CARD methodology can be found in the “CARD Rule Sets” in Supporting information S1. This innovative monitoring tool can aggregate data from each client within a clinical practice to establish percentages of clients who are compliant with medications prescribed during treatment. The analysis also detects unexpected illicit drug use (non- abstinence) in patients tested, relative to expected reported drug use and aggregates that data.

Expected or not expected patient behaviors guide treatment plans and measure outcomes. Treatment decisions cannot be determined based on the simplistic nature of a positive or negative drug test. The basis of the CARD methodology is that drugs present in the body, whether prescribed, or self-reported, have been scientifically proven to exhibit specific conditional results on drug tests. Because drugs can metabolize into other reportable substances, the possibility exists that test results can be open to misinterpretation. CARD engages the patient's prescriptions and/or self-reported drug abuse, their drug test results, and the correlation of thousands of scientifically valid test result conditions to link the ingestion of drugs and subsequent results into classifications of ‘Expected’, ‘Not Expected’, or ‘Alerts’ for physicians. (**See Supporting information S1**). Although we are focused on FDA approved anti-addiction drugs because of duel diagnosis the following drug classes tested included: anabolic steroids, antidepressants, hallucinogens, inhalants, muscle relaxants, opioids, psychostimulants, psychotropic's, sedatives/hypnotics/depressants.

### Statistical Analyses

Statistical analyses were conducted using the discrete contingency analyses, the two-level binomial logistic regression model, and the Generalized Linear Mixed Model (GLMM) in the R package version 2.15.0. The Fisher's exact test was used to evaluate differences in adherence to treatment medications and abstinence rates according to the type of treatment (in-patient *vs*. out-patient), level of care and differences in compliance and abstinence rates in 2010 compared to 2011. We also analyzed a subset of 511 patients representing 17.5% of the entire data set for trend relating to longitudinal urine screens up to 52 times for both compliance and abstinence. It is noteworthy that each and every statistical analysis herein comes with a controlled Type-I error rate.

#### Distribution of Subjects and Specimens Tested

In this analysis data was derived from 10,570 patients at treatment centers across six eastern states of America [Maryland (MD), Maine (ME), North Carolina (NC), Rhode Island (RI), South Carolina (SC) and Vermont (VT)]. The sample size is largely skewed to NC and VT, while SC represents less than 1% of the whole sample (Table 1).

**Table 1:** Distribution of participants by state.

State	Sample Size
Maryland (MD)	218 (7.5)
Maine (ME)	359 (12.3)
North Carolina (NC)	833 (28.5)
Rhode Island (RI)	542 (18.6)
South Carolina (SC)	28 (1.0)
Vermont (VT)	939 (32.1)
**Total**	2919

Although initially there was data from a total 84,206 specimens, to ensure uniformity only first and last specimens tested were used in the analysis. As detailed below in a number of cases the first specimen was obtained in year 2010 while the last specimen was collected in 2011. Of 21,140 urine specimens analyzed across two years (2010 to 2011) the dataset was further filtered to include a total of 2,919 patients who were on at least one prescription medication (*n* = 2,919; total 5838 specimens). The data was stratified over five different levels of care each with the minimum number of calendar days between the first and last urine specimens analyzed (Table 2). The distribution of the number of calendar days between the first and last urine samples is heavily right skewed ranging from 15 days to 717 days. The median is 176 days with the inter-quartile range of 281 days. The sample mean and sample standard deviation are 226.2 days and 171.0 days, respectively.

**Table 2:** Patient level of care and minimum days between samples.

Level of Care	Minimum Days between Samples
In-Patient (IP)	21
Residential facility (RES)	30
Intensive Out-Patient (IOP)	15
Out-Patient (OP)	30
Opiate Treatment Program (OTP)	30

The patient distribution across two modalities and five levels of care is presented in Table 3. The sample consisted of the out-patients (OP) 96%, of which one-third was in the opiate treatment program (OTP). It was found that 11.6% of the patients (*n* = 338) had both the first and last urine specimens collected in 2010 while 44.9% (*n* = 1311) first and last collected in 2011. The rest of 43.5% (*n* = 1270) had the first urine specimen collected in 2010 and the last specimen collected in 2011. All the patients were taking at least one prescription medication and 18.0% of the patients (*n* = 525) were found to be on more than one prescription drug. Definitions of the terms used include; *compliance* being when each and every reported prescription drug was detected, *both* refers to the *first* and *last* urine samples together and *abstinence* means that no analytes that could not be attributed to a reported prescription were detected in urine samples tested. The PATH Foundation IRB reviewed the protocol and waived further approval due to post hoc analysis.

**Table 3:** Distribution of participants over modality and level of care.

Modality	Sample Size	Level of Care	Sample Size
In-Patient	116 (4.0)	IP	41 (1.4)
		RES	75 (2.6)
Out-Patient	2803 (96.0)	IOP	340 (11.6)
		OP	1558 (53.4)
		OTP	905 (31.0)
**Total**	2919	**Total**	2919

IP-In-patients; RES = Residential facility; IOP = Intensive outpatients, OP =  Outpatient; OPT = Opiate treatment programs.

## Results

### Compliance

From both first and last urine specimens, 67.2% of the patients (n = 1963; *P*<.001) had every treatment medication found in both urine samples, hence were “compliance both”. Every treatment medication was found in the first urine sample, 77.9% of the patients (n = 2274; *P*<.001) hence “compliance first”, while, 78.4% of the patients (n = 2289; *P*<.001) had every treatment medication found in the last urine sample, hence “compliance last”. Over the course of the first and last urine specimen collections, it was found that 10.9% of the patients (n = 319) were not complying at all. Improvement in compliance was shown in 11.2% of the patients (n = 326) who did not comply at first but complied at last. However, 10.7% of the patients (n = 311) showed a deteriorating compliance behavior by complying at first but not complying at last urinalysis.

### Abstinence

For the measurement of abstinence, 39.2% of the patients (n = 1143; *P*<.001) had no substance of abuse detected in either of the urine samples, hence “abstinence both”. Moreover, 54.0% of the patients (n = 1577; *P*<.001) had no substance of abuse found in the first urine sample, hence “abstinence first”, while 57.3% of the patients (n = 1672; *P*<.001) had no substance of abuse found in the last urine sample, hence “abstinence last”. Over the course of the first and last urine specimen collections, it was found that 27.9% of the patients (n = 813) were not abstinent at all. Improvement in abstinence was shown in 18.1% of the patients (n = 529) who were not abstinent at first but abstinent at last. The abstinence of 14.9% of the patients (n = 434) deteriorated as they were abstinent at first but not abstinent at last urine drug test.

### Contingency Analyses

The rates of compliance and abstinence across the six eastern states are presented in Table 4. Overall, statistically significant differences were found in the compliance rates as well as the abstinence rates among the six states. That is, ME and NC exhibited the highest compliance rates of over 80%, while SC gave the lowest rate of below 50%. SC and MD, however, showed the highest abstinence rates while ME and RI had the lowest. Illicit drug use during treatment for each state differed whereby: MD = 50.9%; ME = 65.5%; NC = 58.6%; RI = 63.8%; SC = 50.0% and VT = 62.0%.

**Table 4:** Compliance and abstinence rates of participants across the six U.S. eastern states.

Eastern States	Comp B n(%)	Comp F n(%)	Comp L n(%)	Abs B n(%)	Abs F n(%)	Abs L n(%)
**MD**	103(47.3)	127(58.3)	138(63.3)	107(49.1)	145(66.5)	142(65.1)
**ME**	290(80.8)	315(87.7)	326(90.8)	124(34.5)	184(51.3)	184(51.3)
**NC**	690(82.8)	719(86.3)	788(94.6)	345(41.4)	462(55.5)	503(60.4)
**RI**	247(45.6)	349(64.4)	310(57.2)	196(36.2)	262(48.3)	293(54.1)
**SC**	9(32.1)	13(46.4)	13(46.4)	14(50.0)	18(64.3)	20(71.4)
**VT**	624(66.5)	751(80.0)	714(76.0)	357(38.0)	506(53.9)	530(56.4)
**χ^2^**	292.87	179.26	355.14	17.94	23.74	18.97
***p*** **-value**	<.001	<.001	<.001	.003	.002	.002

MD = Maryland; ME =  Maine; NC =  North Carolina; RI =  Rhode Island: SC =  South Carolina; VT = Vermont. Comp = Compliance; Abs =  Abstinence. B = Both; F = First; L = Last.

The rates of compliance and abstinence over the patients' modality are presented in Table 5. From Fisher's exact tests, there is statistically significant evidence to conclude that the out-patients adhered to treatment medications better than the in-patients (*P*_both_<.001; *P*_first_ = .005; *P*_last_<.001). On the other hand, the abstinence rates were similar between the out-patients and the in-patients except for the case of “abstinence last” where the out-patients showed higher tendency of abusing drugs compared to the in-patients (*P*_last_ = .01).

**Table 5:** Compliance and abstinence rates by participants according to treatment modality.

Modality	Comp B n(%)	Comp F n(%)	Comp L n(%)	Abs B n(%)	Abs F n(%)	Abs L n(%)
In-Patient (IP)	56 (48.3)	78 (67.2)	73 (62.9)	51 (44.0)	60 (51.7)	80 (69.0)
Out-Patient (OP)	1907 (68.0)	2196 (78.3)	2216 (79.1)	1092 (39.0)	1517 (54.1)	1592 (56.8)
**χ^2^**	19.74	7.98	17.12	1.17	0.26	6.74
***p*** **-value**	<.001	.005	<.001	.28	.61	.01

Comp = Compliance; Abs = Abstinence. B = Both; F = First; L = Last.

The rates of compliance and abstinence over the patients' level of care are presented in Table 6. Overall a statistically significant difference in the compliance rates as well as the abstinence rates were found among the five levels of care in this study. The patients in the opiate treatment program (OTP) exhibited the highest compliance rates of over 85%, while those in the residential facility (RES) gave the lowest rate of below 65%. The patients in the residential facility (RES), as well as the intensive out-patients (IOP), however, showed the highest abstinence rates while the out-patients (OP) gave consistently low rates.

**Table 6:** Compliance and abstinence rates by participants according to level of care.

Level of Care	Comp B n(%)	Comp F n(%)	Comp L n(%)	Abs B n(%)	Abs F n(%)	Abs L n(%)
IP	22 (53.7)	30 (73.2)	26 (63.4)	14 (34.2)	16 (39.0)	26 (63.4)
RES	34 (45.3)	48 (64.0)	47 (62.7)	37 (49.3)	44 (58.7)	54 (72.0)
IOP	245 (72.1)	281 (82.7)	276 (81.2)	166 (48.8)	228 (67.1)	218 (64.1)
OP	893 (57.3)	1117 (71.7)	1072 (68.8)	553 (35.5)	784 (50.3)	838 (53.8)
OTP	769 (85.0)	798 (88.2)	868 (95.9)	373 (41.2)	505 (55.8)	536 (59.2)
**χ^2^**	222.22	103.78	266.66	27.41	37.38	22.94
***p*** **-value**	<.001	<.001	<.001	<.001	<.001	<.001

IP =  In-Patient; RES = Residential facility; IOP =  Intensive Out-Patient; OP =  Outpatient; OTP =  Opiate Treatment Program. Comp = Compliance; Abs = Abstinence. B = Both; F = First; L = Last.

### Comparison 2010 and 2011

In order to make an annual comparison and examine any trend of the compliance rates and the abstinence rates over time, the dataset was grouped by the year of the collected urine samples. This is important to determine since we hypothesized that familiarity by the staff in using CARD between 2010 when it was first initiated and 2011 one year of experience might help to enhance both compliance and abstinence in respective patients. As already mentioned, 11.6% of the patients (n = 338) had both the first and last urine specimens collected in 2010 while 44.9% (n = 1311) had them collected in 2011. Although no statistically significant difference (each *P*>.30) was found in the compliance rates between 2010 and 2011, Fisher's exact tests revealed that there was statistically significant improvement in the abstinence rates in 2011 compared to 2010 (*P*_both_ = .04; *P*_last_<.001).

Regression analyses revealed that over the course of two urine specimen collections, 15.1% of the patients (n = 51) were not complying at all in 2010, while 14.0% of the patients (n = 183) were not complying at all in 2011. In 2010, 60.1% of the patients (n = 203) were continuously complying, and in 2011, 61.5% of the patients (n = 806) were continuously complying. Improvement in compliance was shown in 11.8% of the patients (n = 40) who did not comply at first but complied at last in 2010. This percentage increased to 13.2% (n = 173) in 2011. In 2010, 13.0% of the patients (n = 44) complied at first but did not comply at last, showing a deteriorating compliance behavior. However, this percentage dropped to 11.4% (n = 149) in 2011. Nevertheless, these changes over time were not found to be statistically significant (*P* = .72).

In addition, over the course of two urine specimen collections, it was found that 35.2% of the patients (*n* = 119) were not abstinent at all in 2010, and the percentage dropped to 28.3% (*n* = 371) in 2011. In 2010, 32.8% of the patients (*n* = 111) were continuously abstinent, and in 2011, the percentage increased to 38.0% (*n* = 498). Improvement in abstinence was shown in 15.1% of the patients (*n* = 51) who were not abstinent at first but were abstinent at last in 2010. The percentage increased to 20.4% (*n* = 268) in 2011. In 2010, 16.9% of the patients (*n* = 57) were abstinent at first but were not abstinent at last, a sign of deteriorating abstinence behavior. This percentage dropped to 13.3% (*n* = 174) in 2011. These changes over years were found to be statistically significant (*P* = .005).

Furthermore we evaluated the association between the compliance and the abstinence measured in this study through odds ratios (OR) and the corresponding 95% confidence intervals. There is statistically significant evidence to show that a compliant patient is more likely to be abstinent during treatment compared to a non-compliant patient. In particular, “abstinence both” is more likely for a patient in “compliance both” than not (OR = 1.34; *P*<.001). Also, “abstinence first” is more likely for a patient in “compliance both” than not (OR = 1.19; *P*<.001), and “abstinence both” is more likely for a patient in “compliance first” than not (OR = 1.17; *P* = .05). Lastly, “abstinence first” is more likely for a patient in “compliance first” than not (OR = 1.29; *P* = .002). It seems that “abstinence last” (or “compliance last”) does not have a statistically significant association with “compliance both/first/last” (or “abstinence both/first/last”) (each *P*>.30).

### Longitudinal Analysis

Following our comparison of data collected in 2010 versus 2011 to further elucidate these interesting findings we looked at urine samples at every time point in a randomly selected subset of patients from 2011.

**Figure 1:**
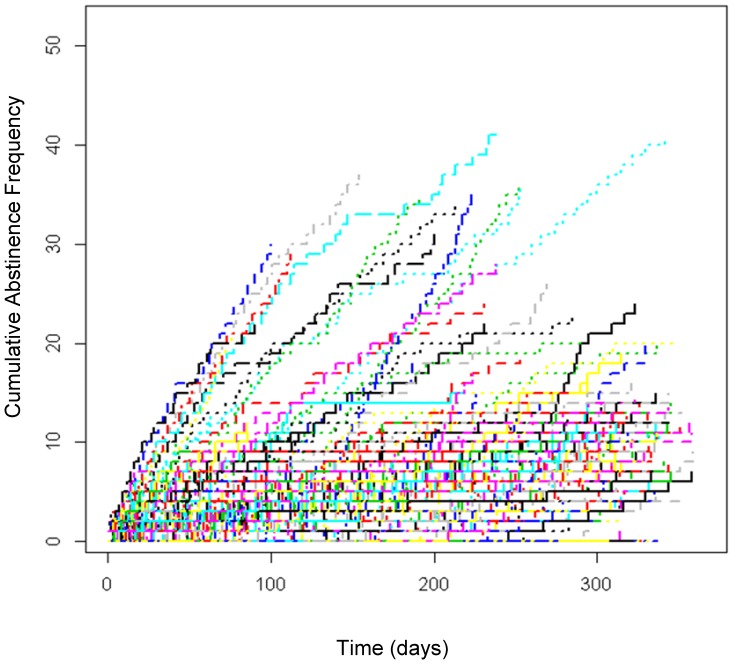
Cumulative Abstinence Frequency over Time.

Figure 1 below describes the longitudinal trend of the abstinence of a subset of the patients (*n* = 511; 17.5%) after removing 56 patients who had only one urine specimen from a pool of patients. Their urine samples were collected in several occasions (up to 52 times) in 2011 and analyzed using CARD. In general, a statistically significant upward trend was observed overall (*p* = 2.353×10^−8^), which implies improved abstinence rates over time.

**Figure 2:**
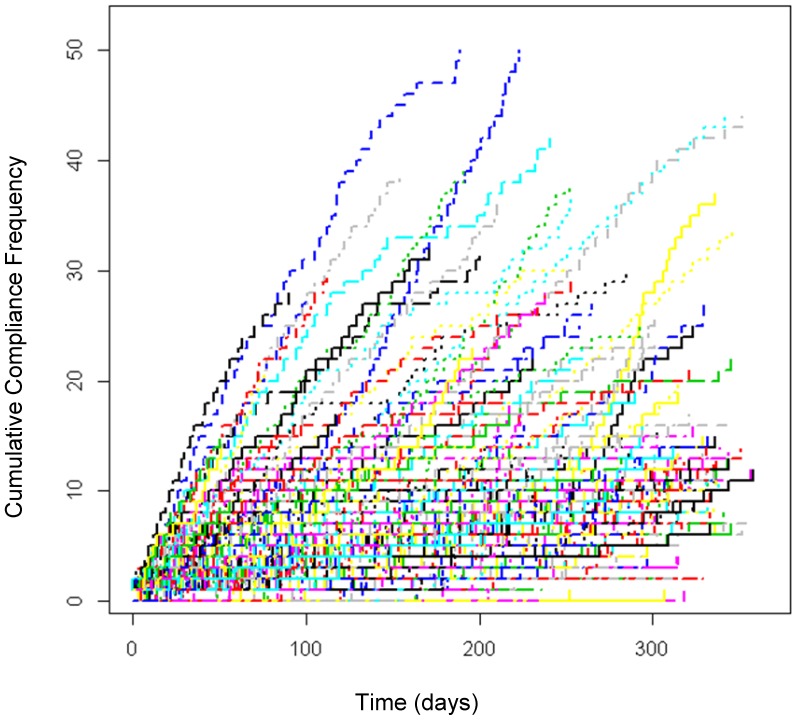
Cumulative Compliance Frequency over Time.

The selected subset of the patients also showed 100% compliance to at least one reported prescription drug. Hence, Figure 2 below describes the longitudinal trend of the improved compliance of the same subset of the patients (*n* = 511; 17.5%) after removing 56 patients who had only one urine specimen from a pool of patients. Their urine samples were collected on several occasions (up to 52 times) in 2011 and analyzed using CARD. Again, in general, a statistically significant upward trend was observed overall (*p* = 2.200×10^−16^), which implies improved compliance rates over time. This upward trend was found to be even stronger than the trend of abstinence.

### Drugs of abuse used by non-abstinent patients

The primary drugs abused by the patients (*n* = 1776) who were not abstinent during treatment are given in Table 7. Almost 23% of the non-abstinent patients used psychostimulants, 25% used benzodiazepines, 32% used cannabinoids, and 38% used opioids during treatment. Use of nicotine was not considered in this analysis. The odds ratios (OR) with the corresponding 95% confidence intervals in Table 5 estimated any association between the abused drug and the compliance to the prescription medications by non-abstinent patients. As large *p*-values indicate, there were no statistically significant associations between the drugs abused and the compliance status in most cases. Compared to a non-compliant patient, it was found that a compliant patient was marginally less likely to abuse opioids (*P* = .07), cannabinoids (*P* = .04), and ethanol (*P* = .07) during the treatment. On the other hand, it was also revealed that a compliant patient was more likely to abuse benzodiazepines compared to a non-compliant patient during the treatment (*P* = .004).

**Table 7:** Primary drugs abused by non-abstinent patients and association to compliance (*n* = 1776).

Drug Class	N (%)	OR	95% C.I.	*p*-value
Psychostimulants	403 (22.7)	0.93	(0.74, 1.18)	.56
Hallucinogens (PCP, LSD, etc.)	4 (0.2)	0.54	(0.08, 3.87)	.54
Inhalants (hydrocarbons)	0 (0.0)	–	–	–
Opioids (analgesics, antitussives, cough suppressants, opiate antagonists)	676 (38.1)	0.83	(0.68, 1.02)	.07
Benzodiazepines (anti-anxiety)	442 (24.9)	1.40	(1.11, 1.77)	.004
Amphetamines	135 (7.6)	0.83	(0.58, 1.19)	.31
Cannabinoids	563 (31.7)	0.81	(0.66, 0.99)	.04
Ethanol	299 (16.8)	0.79	(0.61, 1.02)	.07
Barbiturates	41 (2.3)	0.69	(0.37, 1.29)	.24

Unexpected drug use during treatment in this very large cohort was significant (*p*<0.0001), differed across six eastern states and was significantly greater in non-residential treatment than in residential treatment (*χ* ^2^ = 6.74; *p* = 0.0094; OR = 1.69). From Fisher's exact tests, there is statistically significant evidence to conclude that the out-patients adhered to treatment medications better than the in-patients (*P*_both_<.001; *P*_first_ = .005; *P*_last_<.001). On the other hand, the abstinence rates were similar between the out-patients and the in-patients except for the case of “abstinence last” where the out-patients showed higher tendency of abusing drugs compared to the in-patients (*P*_last_ = .01). The importance of the last urine tested is underscored by revealing the significant difference between in-patients and out-patients, eliminating guessing and providing real quantification of this issue.

## Discussion

Although most clinicians would not be surprised that this analysis of CARD data was not consistent with the null hypothesizes, this is the first report in the chemical dependency literature that quantifies non-adherence to prescribed medication during treatment and the first that found objective evidence of significant drug abuse in recovery (especially during treatment periods). This finding is not surprising especially when we consider the genetic antecedents of addiction and their role in relapse. Recently, Dahlgren [37] found that, 89% (16/18) carriers of the DRD2 A1 allele, reported relapse in contrast to 53% (17/32) in the non-carriers (*p* = 0.01; odds ratio  = 7.1). There is also growing recognition nationally that self-report of drug use is often unreliable. Comings *et al.* [38] associated the dopamine D2 receptor gene A1 allele, with immature defense style (lying) responses to a defense-style questionnaire in patients attending addiction treatment. Certainly, genetic factors coupled with potential fear of punitive measures leads to unreliable self- report of drug use in agreement with the present analysis.

According to Katz and Fanciullo, [39] although there is a lack of accepted diagnostic criteria for medication misuse in patients with chronic pain, awareness of a patient's inappropriate use of illicit drugs or medications, is important in good patient management. It has also been suggested that as the use of opioids for chronic pain increases so does the risk for drug non-adherence, associated drug abuse, potential addiction, and other abnormal drug-related behaviors. McCarberg [30] states that cost-benefit studies suggest that liquid chromatography tests (LUTs) could reduce the costs associated with non-adherence in chronic pain therapy, up to 14.8-fold. Opioids in this present study were indeed the drugs that demonstrated best compliance and were most abused by non-abstinent patients.

Urine drug testing is necessary to determine treatment outcomes and compliance. Input from prescription monitoring programs such as CARD, together with interviews with spouses and medical record reviews may be used to improve patient management and clinical interactions. The finding that abstinence rates improved between 2010 and 2011 and longitudinally during 2011 may be explained by increased honesty in clinical interactions as clinicians gained experience in the use of CARD.

McLellan *et al.* [40] pointed out that relapse rates for addictions are similar to other chronic illnesses, and like diabetes and hypertension, are dependent in-part on adherence to treatment medication. The study finding that compliant patients are more likely to be abstinent during treatment also supports the value of determining non-adherence to medications, which can associate with relapse.

That in-spite of compliance to treatment medications there is a significant unexpected drug use in this large cohort emphasizes the need to find effective anti-relapse treatment modalities to attenuate the known brain-reward circuitry impairment that underpins addiction as defined by ASAM, 2011. In fact, Nora Volkow, irrefutably argues for the development a dopamine D2 agonist that over the long-term up-regulate rather than down regulates these receptors and could provide anti -drug craving and relapse-prevention [41].

The Center for Substance Abuse Treatment already strongly advocates for the use of drug urine testing by treatment program's [28]. Given this context, it would be useful to conduct outcome analyses to further understand why and how some patients and treatment programs are able to get good outcomes and to promote the wide-spread use of practices that successful programs and clinicians employ like, for example, brain reward circuitry balancing especially dopamine up-regulation as observed in epigenetics [42]. Most recently, using genome-wide DNA methylation and gene expression mapping, Zhang observed that in the pre-frontal cortices of deceased alcoholics, compared to matched controls, powerful epigenetics effects impact 126 genes on dopaminergic pathways [43].

## Conclusion

As the very first observational study done in this area, we have identified multiple issues and caveats in our analysis. A limitation of this analysis of pre and post treatment changes is the absence of covariates and lack of systematic collection of urine samples between the first and last sample for each patient. Features of the clinical course of treatment should be considered in the design of future analyses, for example, the first urine collected on admission may be expected to be positive for abused drugs in many programs, while, the last urine might be a trigger for termination of treatment.

Further investigation of this novel CARD database may answer more questions in addition future research to expand the longitudinal aspect of the dataset is needed. This will help to identify any trend or pattern of compliance and abstinence rates over a longer period of time under a systematic and controlled design. This study provides strong objective evidence of non-compliance to treatment medications by a cohort of SUD patients. Additionally, significant drug abuse during treatment was observed across the six states. As expected a large number of patients continue to partially meet treatment expectations with over 60% continuing their struggle with drug abuse.

We found evidence for the effectiveness of substance abuse treatment and based on our longitudinal analysis improvement in outcome for many patients when monitoring both compliance and abstinence are routinely incorporated into treatment. However, albeit there are certain limitations, in that our findings related to a subset of (n = 511) 17.5% of the entire data set; they certainly show a very significant trend for improvement in both compliance and abstinence during treatment.

The analysis of first and last urines found that compared to non-compliant patients, compliant patients were less likely to abuse opioids, cannabinoids, and ethanol during treatment. However, compliant patients were significantly more likely to abuse benzodiazepines. Compliance was significantly higher in residential than in non-residential treatment facilities. Moreover, in 2010, 16.9% of the patients (*n* = 57) were abstinent at first but were not abstinent at last, a sign of deteriorating abstinence behavior. This percentage dropped to 13.3% (*n* = 174) in 2011. This improvement over years was statistically significant however requires confirmation and further exploration by our laboratory and other researchers.

Information from this study does provide some quantitative data both about compliance to FDA approved treatment medications and abstinence from psychoactive licit and illicit drugs of abuse. Additional research is required to further test the clinical utility of this novel tool. These important quantitative findings should impact the clinical issues related to both compliance and abstinence in chemical dependency programs based on medicine assisted therapeutics. Notably, both compliance to prescription medication and abstinence from drugs of abuse was found in many patients supporting the effectiveness of treatment. These findings are in agreement with Reisfield *et al.* who proclaimed that drug urine testing is an invaluable resource for primary care [44]. In fact our longitudinal analysis of a subset of patients albeit, independent of level of care revealed significant improvements in both abstinence and compliance whereby compliance was robust. Future studies are being planned to include level of care in longitudinal analyses in a larger cohort.

While encouraging especially when one considers the positive results in our subset longitudinal analyses, addiction is a chronic disease and overall these results indicate the need for novel therapeutic targets and a paradigm shift in both in-patient and out-patient treatment tactics based on additional research and outcome studies.

## Supporting Information

Supporting information S1 **CARD Rule Sets.**
(DOCX)
