# Supplementary material for: Systematic Evaluation of “Compliance” to Prescribed Treatment Medications and “Abstinence” from Psychoactive Drug Abuse in Chemical Dependence Programs: Data from the Comprehensive Analysis of Reported Drugs
Source: PLoS One. 2014 Sep 23;9(9):e104275. doi: 10.1371/journal.pone.0104275 (PMC4172565; doi:10.1371/journal.pone.0104275)
Supplement: Supporting information S1 — CARD Rule Sets. (DOCX) [file pone.0104275.s001.docx]

**Text S1. Comprehensive Analysis of Reported Drugs CARD™ Rule Sets and Detections**

The CARD™ engine addresses issues of metabolism as well as contaminants in the production processes of some formulations. It addresses multi-faceted scenarios within and across drug classes, often involving the state of multiple analytes in order to reach a conclusion for each self-reported or prescribed drug. It evaluates thousands of rule sets to determine if the statement associated with each rule set is applicable to the specimen test results and reported drugs being analyzed. Each rule set has one or more scientific rules associated with it. Each rule is either analyte-based or based on drug class, and may address detection, prescription, and ingestion - or the lack thereof. The rules are then bound to a rule set that is applicable only when every rule in the rule set evaluates to true. Each rule set requires a scientific reference, a flag (Expected, Not Expected, Alert) and a descriptive comment. The rule sets that are valid as a result of the analysis are published with the lab result report, and easily identify for the clinician whether the patient's state is expected or not expected. Aggregating the analysis results creates objective data relating to patient behaviors and outcomes, as well as information to target which substances show the highest level of reported (or unreported) use.

We further describe herein how the CARD™ message engine determines which statements to display in the CARD™ report produced for a specific specimen, test order, and set of treatment-related and self-reported drugs. A CARD™ message is reported in an analysis when each and every condition in the set of all conditions defined for that message evaluates to true. The set of all conditions defined for a message is called a **condition set**. Each condition in a condition set is composed of a **condition object**, a **condition type**, and a **condition code**. Additionally, if the condition describes an analyte test result, a test **methodology** also is specified. A **condition object** is currently defined to be a reported drug (either treatment-related or self-reported) or an analyte test result. A **reported drug** is the active ingredient in a prescription drug preparation or self-reported drug reported to have been consumed by the patient. An **analyte test result** is a qualitative interpretation of a test result. This qualitative interpretation must be “Detected” or “Not Detected” for a proper evaluation to occur. The **condition type** determines whether CARD™ evaluates analyte test results or the list of consumed drugs for the condition object. When the condition type specifies analyte test, this means the condition object is considered to be an analyte test result. In this case, the CARD™ message engine evaluates the analyte test result to determine whether the condition is true. When the condition type specifies reported drug, this means the condition object is considered to be a reported drug. In this case, the condition evaluates to true or false based on the existence of the specified drug in the reported drug list associated with the urine sample at time of collection. The **condition code** defines whether the condition object associated to the condition is required to be present, required to be absent, or, if the condition type specifies analyte test, whether the analyte test was even performed. This characteristic allows CARD™ to define a condition based on the absence or presence of a reported drug. It also allows CARD™ to define a condition based on whether an analyte test resulted in Detected or Not Detected, or whether the test was run at all. Most of the CARD™ messages require the presence of a reported drug or analyte plus the absence of one or more reported drugs or analytes.

The **methodology** attribute allows for CARD™ to differentiate between analyte test results produced by methodologies of varying specificity and sensitivity. For example, imagine a statement that should only be displayed when an EIA-based analyte test has resulted to Detected and when no other more sensitive or specific test for that analyte (e.g., a liquid chromatography-tandem mass spectrometry [LC-MS/MS]-based test) has also been performed. The condition set for that statement will be composed of a condition that specifies the EIA-based test for that analyte has resulted to Detected, as well as another condition that specifies that the LC-MS/MS-based test for the same analyte has not been performed. The condition set composed of these two conditions will evaluate to true only when the EIA-based test result is Detected and the LC-MS/MS-based test was not performed.

A **condition set** is defined as one or more conditions. When every condition in that condition set evaluates to true, the message associated with that condition set is displayed in the CARD™ report. Thus, the CARD™ message engine compiles a list of all condition sets for which any condition from that condition set evaluates to true for the sample being analyzed. For each condition set in that list, it then counts the number of reported drug conditions that evaluate to true and the number of analyte test result conditions that evaluate to true. It then compares the true condition counts calculated for each condition set with the number of conditions defined for that condition set. For each condition set in the list, when the number of reported drug conditions evaluated to true, plus the number of analyte conditions evaluated to true, match the number of reported drug conditions plus analyte conditions defined for that condition set, the CARD™ message engine evaluates the condition set to true and displays the message associated with that condition set.

Similarly, the CARD™ message engine evaluates a condition. A condition associates a condition object with a condition type, condition code, and optionally, a methodology. The condition type determines whether we evaluate the condition by examining the analyte test results or the reported drug list. For an analyte test result condition, the condition code determines whether the analyte test resulted to Detected, Not Detected, or whether the test was even performed. For a reported drug condition, the condition code determines whether we look for the presence of the reported drug or the absence of the reported drug in the reported drug list.
